# Linking environmental risk factors with epigenetic mechanisms in Parkinson’s disease

**DOI:** 10.1038/s41531-023-00568-z

**Published:** 2023-08-25

**Authors:** Maria Tsalenchuk, Steve M. Gentleman, Sarah J. Marzi

**Affiliations:** 1 UK Dementia Research Institute, Imperial College London, London, UK; 2 Department of Brain Sciences, Imperial College London, London, UK

**Keywords:** Parkinson's disease, Epigenetics, Risk factors

## Abstract

Sporadic Parkinson’s disease (PD) is a progressive neurodegenerative disease, with a complex risk structure thought to be influenced by interactions between genetic variants and environmental exposures, although the full aetiology is unknown. Environmental factors, including pesticides, have been reported to increase the risk of developing the disease. Growing evidence suggests epigenetic changes are key mechanisms by which these environmental factors act upon gene regulation, in disease-relevant cell types. We present a systematic review critically appraising and summarising the current body of evidence of the relationship between epigenetic mechanisms and environmental risk factors in PD to inform future research in this area. Epigenetic studies of relevant environmental risk factors in animal and cell models have yielded promising results, however, research in humans is just emerging. While published studies in humans are currently relatively limited, the importance of the field for the elucidation of molecular mechanisms of pathogenesis opens clear and promising avenues for the future of PD research. Carefully designed epidemiological studies carried out in PD patients hold great potential to uncover disease-relevant gene regulatory mechanisms. Therefore, to advance this burgeoning field, we recommend broadening the scope of investigations to include more environmental exposures, increasing sample sizes, focusing on disease-relevant cell types, and recruiting more diverse cohorts.

## Background

Parkinson’s disease (PD) is a progressive neurodegenerative disorder, clinically characterised by rest tremors, bradykinesia and shuffling gait, in addition to psychiatric disturbances, cognitive decline and autonomic dysfunction^1^. One of the main pathological signs of PD is the selective death of dopaminergic neurons in the substantia nigra pars compacta (SNpc) and subsequent deregulation of the basal ganglia, resulting in aberrant motor control^2,3^. A further hallmark is the development of Lewy bodies and neurites in neurons and processes, respectively^4^. A major component of Lewy bodies is misfolded, highly ubiquitinated α-synuclein (α-syn) protein^4,5^. Further proteins identified in Lewy bodies include neurofilament and αβ-crystallin^6,7^, in addition to other molecular components such as lipid membranes, lysosomal structures and mitochondria^8^. The strongest known risk factor for developing PD is age, with 1–3% of the population over 60 affected by PD, rising to 5% over the age of 85^9^. PD is primarily a sporadic disorder, accounting for 85–90% of cases. However, several genes have been identified that give rise to rare familial forms of PD when harbouring mutations^10^. The most common genetic risk factors include variants in *LRRK2* and *GBA1*, involved in lysosomal and autophagic pathways^11^. In contrast, sporadic PD shows only moderate heritability of 0.19 as estimated by twin studies^12^, with a consistent association with PD risk coming from common variants in *SNCA*, identified by genome-wide association studies (GWAS)^13,14^. This suggests considerable contributions from environmental risk factors. Indeed, several environmental exposures have been explored in association with PD risk, including tobacco smoking, pesticide exposure and traumatic brain injury^15–17^. However, genetics, ageing or environmental exposures have thus far failed to fully explain the mechanisms through which PD risk acts on cellular phenotypes^18^. Integrating established associations between these factors and gene regulatory and epigenetic signatures has the potential to unravel molecular pathways involved in the development and progression of PD. Recently, there has been increasing emphasis on studying epigenetic pathways involved in PD, largely as a potential link between environmental stimuli and genetic information. >90% of single nucleotide polymorphisms (SNPs), and therefore most risk loci, reside in non-coding regions of the genome, likely acting via altering the activity of gene regulatory elements like gene promoters and enhancers^19^. Interpreting non-coding risk variants from GWAS via gene regulatory annotation has proven useful to interpret genetic risk in PD, highlighting, for example, which cell types are primarily affected by genetic risk variants^20,21^.

### Epigenetics

The term epigenetics refers to mechanisms that alter gene activity without changing the genetic sequence^22^. The three fundamental classes of epigenetic mechanisms are DNA modifications, histone modifications and non-coding RNA. The most prevalent and widely researched DNA modification is DNA methylation, wherein a methyl group is added to the C5 position (5mC) on cytosine residues in CpG dinucleotides^23^. CpG-rich stretches of DNA, termed CpG islands, are prevalent in gene promoter regions. Dense DNA methylation at CpG islands is typically associated with tightly packed heterochromatin and subsequent transcriptional repression^24^, although the function of DNA methylation in other genomic regions is less well understood^25^. DNA demethylation is catalysed by ten-eleven translocation (TET) enzymes, which oxidise 5mC to its intermediary hydroxymethylated form (5hmC)^26^. Interestingly, DNA hydroxymethylation has also been shown to be a stable epigenetic mark, thought to be involved in gene regulation^27–29^. Chromosomal DNA is packaged around histones, which are nuclear proteins acting as spools for chromatin. Histone modifications occur at the N-terminal tails of histones, which can alter chromatin structure directly by affecting the local charge of the histone protein and thereby the histone-DNA interactions or by recruiting effector proteins which subsequently change the chromatin conformation^30^. Histone modifications include methylation, acetylation, phosphorylation and ubiquitination and the occurrence and combination of histone modifications found has been linked to functionally distinct genomic regions^31^. Finally, there are multiple classes of non-coding RNA - expressed transcripts, which do not code for proteins. These non-coding RNAs can act on post-transcriptional gene silencing, control transposable elements and direct epigenetic complexes^32^.

### Epigenetics in PD

DNA methylation is the most studied epigenetic mechanism in the context of PD. Aberrant methylation patterns have been found in post-mortem brain^33^, leucocytes^34^, whole blood and saliva^35^ collected from PD patients. Such changes in DNA methylation are predicted to modulate gene expression. Henderson et al. found seven differentially expressed genes associated with 62 differentially methylated CpG sites in the blood of PD patients compared to controls (*n* = 15 cases, *n* = 15 controls), indicating these genes may be regulated by disease-relevant DNA methylation patterns^36^. Another epigenome-wide association study (EWAS) found seven genome-wide significant CpGs associated with cognitive decline and eight with faster motor progression, with the motor progression CpG sites annotated to genes related to synaptic function, Wnt signalling and mitochondrial apoptosis^37^. Kochmanski et al. (2022) reported PD-associated methylation changes in *DJ-1*, *VGLUT2*, *IA-2β* and *NURR1* in an enriched neuronal population from PD post-mortem parietal cortex^38^. Crucially, this was the first genome-wide study of DNA methylation to stratify by cell-type and sex in the analyses.

Global DNA hypomethylation in PD SN samples has been reported, including at the *SNCA* promoter^39,40^, although this finding did not replicate across all studies^41,42^. Similar changes have been observed in brain tissue from patients with PD and dementia with Lewy bodies (DLB)^43^. Key enzymes involved in methylation pathways were also found to be associated with PD. This includes mislocalisation of nuclear DNA methyltransferase 1 (DNMT1) in the cytoplasm of neurons of PD patients^43^ and association of *TET1* variants with PD status^44^. Altered methylation of *CYP2E1* was reported in two studies^45,46^, while dynamic DNA methylation changes were observed in PD patients receiving anti-parkinsonian medication compared to treatment-naïve patients^45^. Two studies showed that global levels of 5hmC are increased in post-mortem cerebellum in PD cases compared to age-matched controls^47,48^, while no change was seen in the cortex or brain stem^48^.

The role of histone modifications in PD has been less extensively studied, however, there is evidence to suggest dysregulated histone acetylation in PD compared to healthy brain samples. One study reported increased acetylation of multiple histone tail residues, with H3K27 showing the most significant change in acetylation^49^. This is noteworthy, as H3K27ac has been shown to be dysregulated in other neurodegenerative diseases, including late-onset Alzheimer’s disease^50–52^. The same study reported widespread region-specific changes associated with PD in post-mortem brain samples. However, of note, the brain tissue investigated was the prefrontal cortex as opposed to the primarily affected midbrain^49^.

### Environmental risk factors in PD

**Fig. 1:**
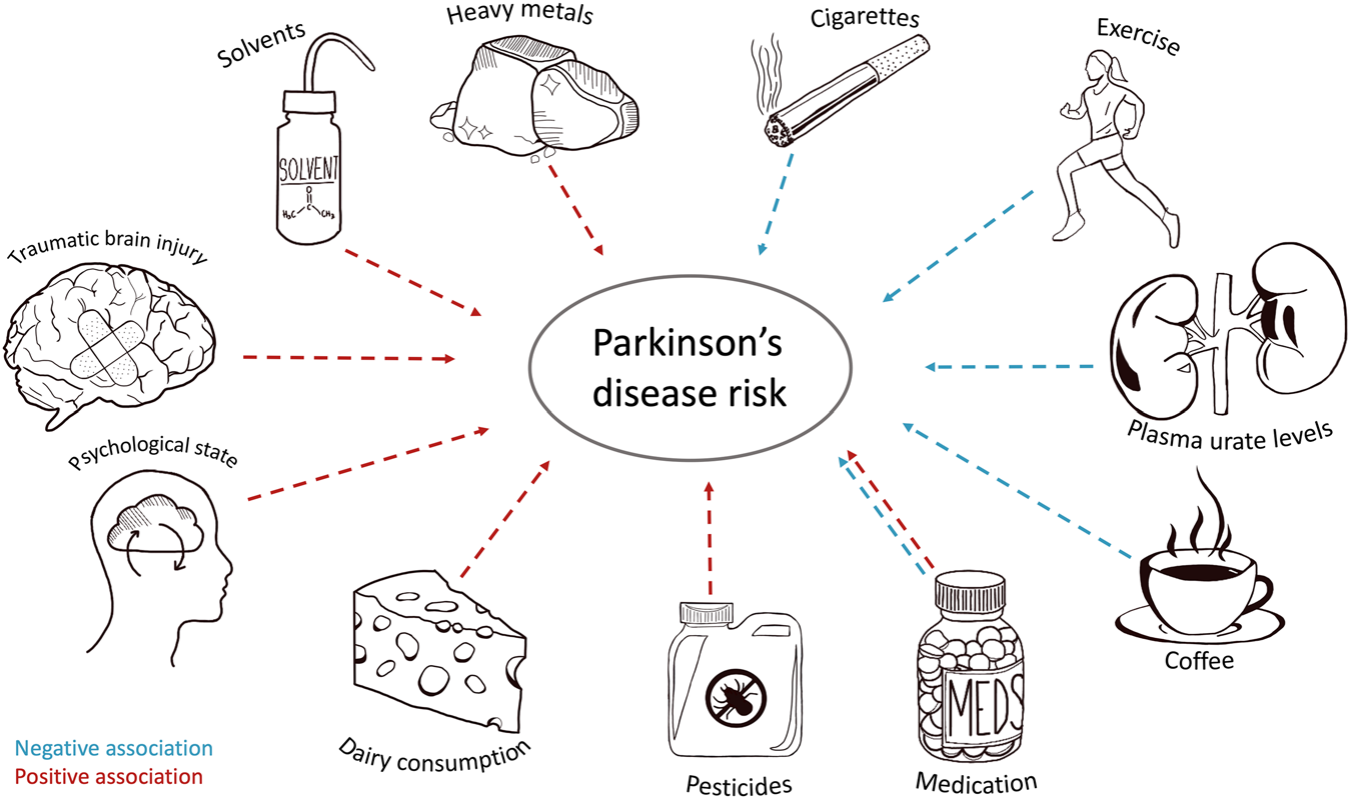
Exposures and environmental risk factors reported to be associated with Parkinson’s disease.

Many environmental exposures have been explored in relation to PD risk, although it is exceedingly difficult to identify those critical and causal in disease aetiology (Fig. 1). Interestingly, there are numerous factors shown to be negatively associated with PD risk. Cigarette smoking is one such factor, which shows a strong negative dose-response relationship with PD risk: the longer and more participants smoked, the less likely they were to develop PD^15^. Coffee drinking is also associated with a lower risk for both disease risk and progression^53^. The presence of caffeine and eicosanoyl-5-hydroxytryptamide (EHT) in coffee could in part explain this association. These compounds enhance the activity of protein phosphatase 2A (PP2A) responsible for dephosphorylation of α-syn, resulting in reduced aggregation^54,55^. Other explanations include the confounding effects of low-risk taking personality traits, caused by low dopamine levels, leading to less smoking and coffee drinking behaviours of people in prodromal stages of PD^56^. A final, relatively novel, theory suggests that caffeine and tobacco change the gut microbiota in ways that mitigate misfolding of α-syn in enteric neurons^57^. Additional negatively correlated factors include the use of ibuprofen^58^, physical exercise^59,60^ and plasma urate levels^61,62^, although often with contradictory evidence^63–65^.

Other environmental factors are associated with an increase in PD risk. Studies suggest exposure to chemicals such as pesticides, herbicides and solvents are implicated in PD. To date, pesticides and herbicides have been more widely researched than other exposures, prompted by structural similarities of the herbicide paraquat^66^ and pesticide rotenone^67^ to the known parkinsonism-causing metabolite 1-methyl-4-phenylpyridinium (MPP^+^)^68^. Although rural living and agricultural jobs have been touted as significant risk factors for developing PD, this appears to be chiefly due to increased exposure to pesticides^69,70^. Living in urban areas with high levels of air pollution has also been linked to an increased risk of developing PD^71,72^. Tanner et al. reported that the risk of parkinsonism increased with the use of pesticides including rotenone, dieldrin and paraquat^70^, a finding consistently reported by several studies^69,73,74^. Welders and people in the mining industry are also thought to be at increased risk of developing PD^75,76^. Supporting this, studies have shown a higher incidence of PD in people exposed to heavy metals. Gorell et al. reported an increased risk of PD in those exposed to copper, manganese and lead^77^. For example, workers in the top quartile for chronic occupational exposure to lead had a twofold risk of developing PD compared to those in the bottom quartile^78^. However, there is conflicting evidence disputing these claims^79,80^. Occupations involving exposure to solvents, such as trichloroethylene (TCE), have also been linked with an increased risk of developing PD^81,82^. Contradicting this, a population-based case–control study showed no correlation between solvent exposure and PD^83^, although it should be noted that, for all mentioned studies, cohorts were small and recall bias (i.e. of duration and concentration of exposure) could skew data^84^. Traumatic brain injury (TBI) has been investigated in relation to PD. Both severe head trauma and mild TBI, i.e. with concussion, significantly increased the risk of developing PD^85,86^. Other reported positive associations with PD risk include consumption of dairy products^87,88^, use of ß-blockers^89,90^, and diagnosis of depression and anxiety^91,92^. Reviews by De Miranda et al.^93^ and Dorsey et al.^94^ offer comprehensive insights into the importance of investigating environmental exposures in Parkinson’s disease (PD).

### Linking environmental risk factors and epigenetic regulation in models of PD

**Fig. 2:**
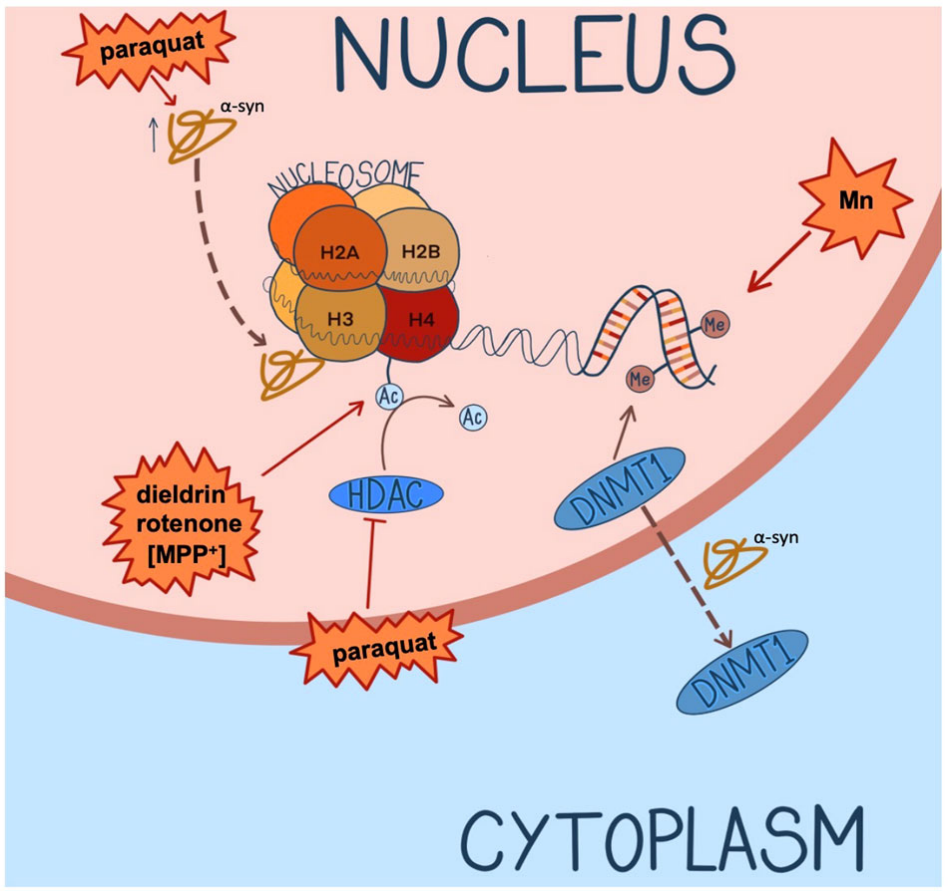
Overview of reported links between environmental risk factors of PD and epigenetic mechanisms. The pesticide paraquat upregulates α-syn and facilitates colocalisation with histones in mouse nigral neurons. Paraquat also increases acetylation of multiple histone residues in SH-SY5Y cells and rat N27 dopaminergic cells, the latter via inhibition of HDAC. MPP^+^ (indicated in brackets as it is not strictly an environmental exposure) and rotenone increase acetylation of histones in SH-SY5Y cells, while dieldrin has the same effect in N27 cells as well as SNpc and striatum of mice. The heavy metal manganese increases methylation of CpG sites in SH-SY5Y cells. In human PD cases and SNCA transgenic mice, α-syn sequesters DNMT1 out of the nucleus, thereby reducing total levels of DNA methylation^43,96–99,107^.

Evidence of the entanglement of environmental factors and epigenetics has been shown in in vitro and in vivo models of PD (Fig. 2). Chemicals, such as 1‐methyl‐4‐phenyl‐1,2,3,6‐tetrahydropyridine (MPTP) and its metabolite MPP^+^, have provided a valuable experimental tool and revealed epigenetic changes in response to neurotoxins. While the MPTP model does not fall under the category of environmental exposure, its administration results in pronounced, selective degeneration of dopaminergic neurons in the midbrain in in vivo models^95^. One study administering MPTP in SH-SY5Y cells, a human-derived neuroblastoma cell line frequently used in PD research, revealed increases in acetylation of multiple histone sites upon exposure to MPP^+^^96^. This was further confirmed in mice exposed to chronic MPP^+^, which showed an increase in acetylation of histones in tyrosine hydroxylase positive (TH+) neurons of the midbrain^96^. The same study reported an increase in histone H2B (Lys15) acetylation in SH-SY5Y cells upon exposure to pesticides rotenone and paraquat. Consistent with this finding, N27 rat dopaminergic cells exposed to paraquat showed a time-dependent increase in acetylation of H3 and H4, seemingly through inhibition of histone deacetylase (HDAC) activity^97^. Interestingly, α-syn was upregulated and shown to colocalise with histones in nigral neurons taken from mice exposed to chronic paraquat, indicating an interaction between α-syn and histones may contribute to PD pathophysiology^98^. Exposure to dieldrin yielded similar results in N27 cells, in addition to increased acetylation of H4 of cells in the SNpc and striatum of mice^99^. An in vivo study, using C57BL/6 mice exposed to 0.3 mg/kg dieldrin, showed differentially methylated CpG sites in regions related to dopaminergic differentiation; namely encompassing *NURR1* and *LMX1B*^100^. Building on human epidemiological data shown previously, in vitro studies have suggested chronic exposure to heavy metals may also play a role in PD. Tarale et al. exposed SH-SY5Y cells to manganese, resulting in an increase of methylation at CpG sites from 70.4% to 73.6%, including hypermethylation of PD-associated genes *PINK1*, *PARK2* and *TH*^101^. Lead exposure has also been linked to PD risk. Parallels between altered epigenetics of PD patients and in vivo models can be seen; decreased H3K9ac is observed in human PD post-mortem primary motor cortex^102^ and is similarly seen in the hippocampus of rats chronically exposed to lead^103^. For a comprehensive review on lead-induced epigenetics in relation to PD see Wang et al.^104^.

Exposure to air pollution has been associated with altered DNA methylation in humans^105^ and has been studied in mouse models: C57BL/6J mice exposed to particulate air pollution exhibited lower H3K9me2/me3 repressive histone marks and displayed increased levels of tau phosphorylation, indicating particulate matter in polluted air may play a role in neurodegeneration via epigenetic mechanisms^106^.

This systematic review aimed to summarise and critically evaluate the existing literature on the link between environmental exposures and epigenetic regulation in PD. While this question has been discussed previously^108^, a structured, systematic approach was lacking. This serves the twofold purpose of assessing the state of the science of the emerging field of environmental epigenetics in PD, as well as the validity of clinical concerns for PD patients arising from such studies. In doing so, we identified gaps in the literature and make recommendations for future studies investigating the interplay of environment and epigenetics in PD.

## Methods

### Protocol and registration

Our systematic literature review was conducted according to the Preferred Reporting Items for Systematic Reviews and Meta-Analyses (PRISMA) guidelines^109^. The protocol for the review was registered on the international prospective register for systematic reviews, PROSPERO, on 8 June 2021 before data extraction was completed (CRD42021257097).

### Search strategy

An exhaustive literature search was conducted on 29 October 2022, using the electronic databases PubMed, Embase, MEDLINE, Web of Science and Scopus. We combined text, and where appropriate, Medical Subject Headings for Parkinson’s disease, related environmental exposures, and epigenetic measures. The search terms for PubMed are shown below:

(“Parkinson Disease”[mh] OR Parkinson*[tiab]) AND (Epigenomics[mh] OR epigenetics[tiab] OR “DNA methyl*“ OR “CpG Islands”[mh] OR demethylat*[tiab] OR histone[tiab] OR “non-coding RNA”) AND (toxicant[tiab] OR solvent[tiab] OR environment[tiab] OR cigarette*[tiab] OR smoking[tiab] OR coffee[tiab] OR caffeine[tiab] OR pesticide[tiab] OR metal[tiab] OR “air pollution” OR contaminant[tiab] OR exposure[tiab] OR ibuprofen[tiab])

Exact search terms for other databases can be found in Supplementary S1.

To reduce penalisation of studies due to absence of technology, articles were limited to those published after 2003 when next-generation sequencing and microarray-based technologies became widely accessible. Only publications that pertained to humans and were written in English were included. The search and filtering process were performed independently by two authors to avoid selection bias.

### Selection criteria

Articles were selected for inclusion in the systematic review if they fulfilled the following criteria:

1. Studies investigating humans only
2. Clinical diagnoses of sporadic PD using recognised diagnostic criteria, including UK Brain Bank and Gelb diagnostic criteria and the Hoehn & Yahr and Unified Parkinson Disease Rating scales
3. Investigations of any type of environmental exposure
4. Investigations of any type of epigenetic marker, including DNA modifications, histone modifications and non-coding RNAs
5. Studies including a control or comparison group
6. Publications in English only
7. Publications published from 2003 onwards

**Fig. 3:**
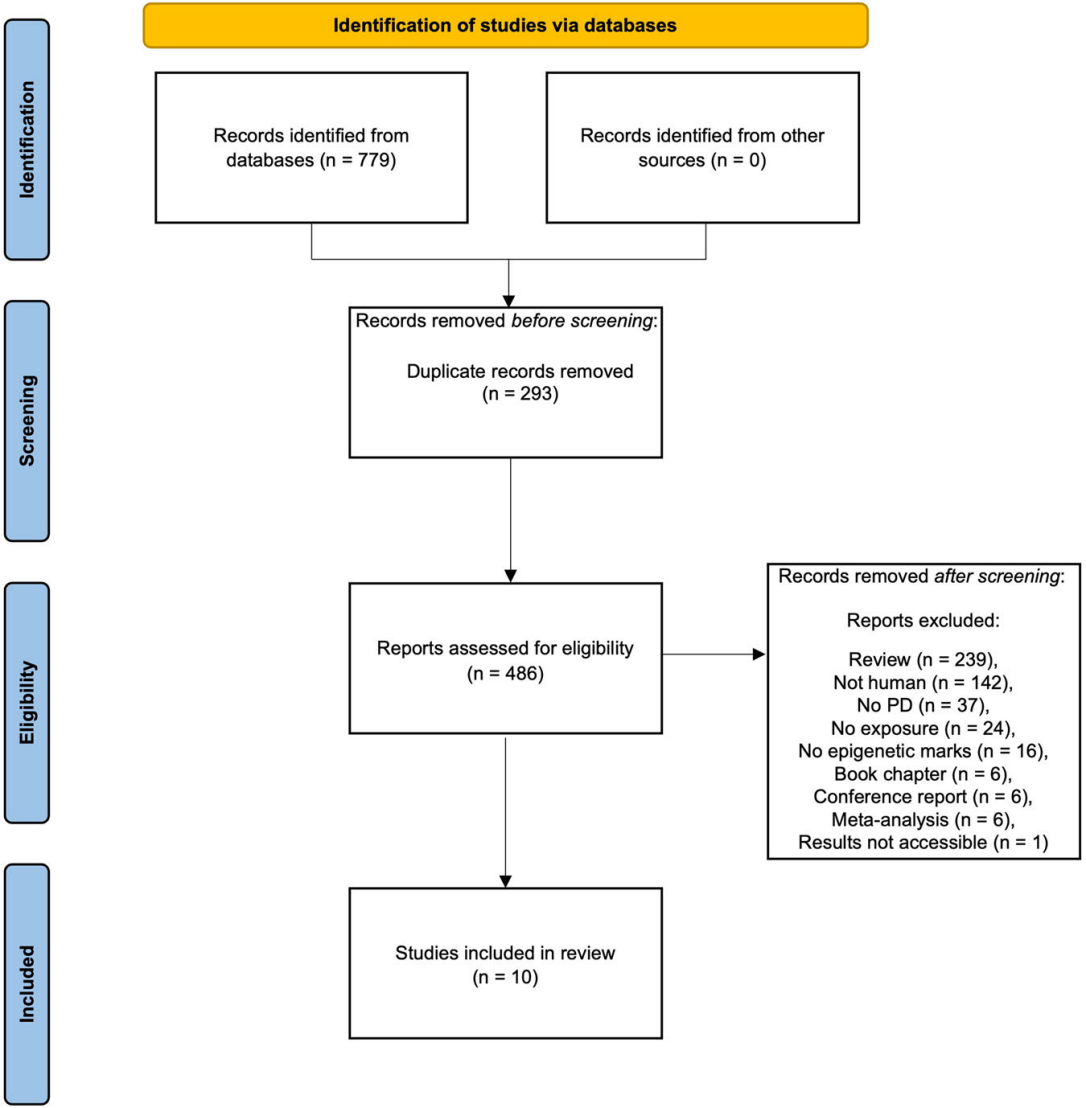
PRISMA flow diagram of study selection.

Articles identified through the search which did not meet the inclusion criteria detailed above were recorded along with their reason for being excluded in Supplementary S2. A flow diagram of the selection process, including the number of articles filtered at each step of the process is shown in Fig. 3.

### Data extraction

The following information was extracted from every identified study:

- Author
- Year of publication
- Rationale for study
- Epigenetic mark investigated and assay method
- Environmental exposure investigated
- Outcome variables (global epigenetic measurement, genome-wide approach, candidate genes)
- Tissue or cell type
- Cohort
- Number of study participants
- Age of study participants
- Matching of cases and controls
- Main results of study
- Statistical analyses
- Covariates
- Exclusion/inclusion criteria applied in the study

Information was recorded by the first author and independently verified by the senior author. Any disagreements were resolved in the discussion. Complete records can be found in Supplementary S3.

### Quality of evidence assessment

A Grading of Recommendations, Assessment, Development and Evaluations (GRADE) framework was used to assess the quality of evidence^110^. Nine criteria were used to assess the study quality: study design, risk of bias, inconsistency, indirectness, imprecision, publication bias, large effect, dose-response and no plausible confounding.

## Results

A total of 468 individual papers were identified by our search terms. 232 were excluded as they were reviews, 138 were cell or animal studies, 36 were not focused on PD, 19 did not study specific environmental exposures, 16 did not test any epigenetic markers, six were book chapters, six were conference reports of previously excluded papers and four were meta-analyses (Fig. 3). We contacted the authors of a relevant conference abstract for additional information on their study, however, this was subsequently removed from our included studies as the results were inaccessible to us. In total, 10 published papers were included in this review (Table 1). To our knowledge, these papers represent all the published studies investigating the impact of environmental exposure on epigenetics in human PD.

**Table 1:** Studies assessing the effect of environmental exposures on epigenetics in human PD cases.

Study	Sample size	Epigenetic measure	Tissue	Exposure	Main results
Chuang et al.^35^	Blood: Controls = 215 (PEG1 cohort); 2100 (WHI cohort), Saliva: Cases = 127 (PEG2 cohort), Controls = 129 (PEG2 cohort)	DNA methylation	Blood, saliva	Coffee consumption	• Hypomethylation of cg21566642 near *ALPPL2* in blood linked to coffee consumption in controls at genome-wide significance • Five CpGs significant at *P* < 5.0 × 10^−6^ (annotated to *BSCL2*, *PODXL*, *CNTN4*, *GPR132* and *ROBO3*) • No significant associations in saliva of PD cases or controls
Coupland et al.^111^	Leucocyte: Cases = 358, Controls = 1084; Brain cohort 1 (cerebellum): Controls = 69; Brain cohort 2 (cerebellum, anterior cingulate cortex, putamen): Cases = 28, Controls = 12	DNA methylation	Leucocytes, post-mortem brain	Vitamin E, PD drugs	• *MAPT* hypermethylation associated with vitamin E levels in leucocyte controls • Reduced *MAPT* methylation with levodopa use in leucocyte PD cases
Paul et al.^115^	SGPD: Cases = 959, Controls = 930; PEG: Cases = 569, Controls = 238	DNA methylation	Whole blood	Lead	• PD status strongly associated with DNA hypermethylation-derived predicted lead levels in the tibial bone, but not the patella bone
Go et al.^113^	Cases = 20 (blood, brain)	DNA methylation	Blood, post-mortem brain	Organochlorine pesticide	• Seven DMPs in PD post-mortem brain with 10+ years plantation work (top two hypermethylated and annotated to *PTGDS* and *PEX19*) • 123 DMPs in blood (top two hypermethylated and annotated to *WNT16* and *ENTPD8*)
Oliveira et al.^112^	Cases = 10, Controls = 9	Global H4 acetylation	Blood	Grape juice and exercise	• Global increase in H4 acetylation in blood of PD patients in response to exercise and grape juice treatment • No differences between exercise alone and exercise plus grape juice
Henderson-Smith et al.^45^	Cases = 189, Controls = 191	DNA methylation	Blood	PD drugs	• 237 DMPs PD patients taking dopamine replacement therapies (between 0.63% reduction and 0.86% increase in DNA methylation per year).
Paul et al.^117^	Blood: Cases = 342, Controls = 238 (PEG1 cohort); Saliva: Cases = 128, Controls = 131 (PEG2 cohort)	DNA methylation	Blood, saliva	Organophosphate (OP) pesticides	• Seven DMPs associated with OP exposure in PD (annotated to *ALOX12*, *HLA*, *HLA-L*, *HLA-DPA1*, *POLR1B* and *HDAC4*).
Castillo et al.^114^	Cases = 97 (high-mining area = 45, low-mining area = 52), Controls = 111 (high-mining area = 52, low-mining area = 59)	DNA methylation	Whole blood	Heavy metal mining	• Lower DNAm levels in controls low mining towns • No difference seen in PD cases
Searles Nielsen et al.^116^	Cases = 49, Controls = 103, Intermediate Controls (UPDRS3 ≥ 6 to <15) = 49	DNA methylation	Whole blood	Manganese	• Inverse association between *NOS2* methylation and welding • CpG site 8329 decreased in methylation with each year of welding exposure
Searles Nielsen et al.^120^	Cases = 292, Controls = 401	DNA methylation	PBMC	Smoking	• No association between LINE-1 methylation and PD in PBMCs • Suggestive association between ever-smoking and PD; strongest in participants with low LINE-1 methylation

*PEG1* Parkinson’s Environment and Genes wave 1, *PEG2* Parkinson’s Environment and Genes wave 2, *WHI* Women’s Health Initiative, *SGPD* System Genomics of PD, *UPDRS3* Unified Parkinson’s Disease Rating Scale.

### Recent body of evidence

We classified the selected studies according to the measured environmental exposures into three groups: three studies investigated heavy metals, two focused on pesticides and the remaining five studies could loosely be grouped under lifestyle factors (Supplementary S3). This category included studies exploring the impact on epigenetics in PD of drugs, coffee consumption, tobacco smoking, dietary vitamin E levels and finally one study on grape juice consumption and exercise.

Studies primarily focused on DNA methylation, with most using the Illumina 450 K HumanMethylation array, while one study used the EPIC array^45^ and another a luciferase-reporter assay^111^. One study measured global H4 acetylation using colorimetric detection^112^. There was some overlap in the cohorts from which data was taken; three studies used the Parkinson’s Genes and Environment Study (PEG1 and PEG2), four generated their own data from participants. Other existing population and PD-specific cohorts, including the Sydney Memory and Ageing Study (MAS), Queensland PD Study, Harvard Biomarker Study, Women’s Health Initiative (WHI), Honolulu Aging Study, Group Health Cooperative and System Genomics of PD (SGPD) were used in the reported studies. Most studies profiled epigenetic signatures in surrogate tissues including whole blood, leucocytes, peripheral blood mononuclear cells (PBMC) and saliva, however, two studies also measured epigenetic markers across multiple brain regions in post-mortem tissue^111,113^. Only four studies corrected for cellular composition within heterogeneous tissues, including one on brain tissue. Only two of the eight studies conducted in peripheral tissue discussed the limitations of using surrogate tissue as opposed to brain tissue. Most studies included participants of mixed sex. However, two studies only included male subjects—which is not unusual as PD is more prevalent in males.

### Heavy metals

Three papers investigated the effect of heavy metal exposure on epigenetics in PD patients, in whole blood samples^114–116^. A candidate gene study investigating DNA methylation of the gene *NOS2*, which encodes inducible nitrous oxide synthase, found an inverse association between *NOS2* methylation in whole blood and parkinsonism among welders (*n* = 93 cases, *n* = 103 controls)^116^. Specifically, methylation at CpG site *NOS2* 8329 decreased with each year of welding exposure. Another study^114^ looked at the effects of heavy metal mining on global DNA methylation comparing whole blood in PD patients and controls from two regions in Chile with different mining exposures. The low mining town contributes 0.7% of mineral production in Chile, while the high mining is responsible for 51%. This study found no difference in DNA methylation between participants with PD from towns with high mining activity compared to low but did see a difference in controls (*n* = 97 cases of which 45 were from a high mining town, *n* = 111 controls of which 52 were from a high mining town). Individuals experiencing low mining exposure had lower DNA methylation than those exposed to high mining activity. Lastly, a publication^115^ investigated genome-wide DNA methylation patterns in the tibia and patella as a biomarker to quantify lead exposure. The authors found that PD status was strongly associated with DNA methylation-derived lead level estimates from the tibia across cohorts (*n* = 959 cases, *n* = 930 controls). However, the patella biomarker showed inconsistent results, with an inverse correlation with PD observed in some cohorts but not others, potentially due to the shorter half-life of lead in the patella, as suggested by the authors.

### Pesticides

Two studies investigated genome-wide DNA methylation profiles in PD in relation to pesticide exposure. Paul et al.^117^ profiled DNA methylation in whole blood samples from PD (*n* = 342) and control participants (*n* = 238), reporting 70 differentially methylated CpGs, of which 27 were associated with organophosphate (OP) exposure. Exposure-associated DNA methylation differences were reported both in PD cases (*P* = 4.11 × 10^−8^) and in controls (*P* = 0.001). Of these, only seven were disease-specific, where associations with OP exposures were solely seen in PD patients. CpG sites associated with OP exposure included those annotated to *ALOX12, HLA*, *HLA-L*, *HLA-DPA1*, *POLR1B* and *HDAC*4—genes involved in lipid mediation, immune response, transcription and epigenetic modification. In contrast to blood, only 14 differentially methylated CpG sites associated with OP exposure were identified in saliva (*n* = 128 cases, *n* = 131 controls). Go et al.^113^ identified seven differentially methylated positions (DMPs) in occipital lobe samples of PD cases with 10+ years of plantation work compared to unexposed cases (*n* = 4 cases, *n* = 13 controls). The top two annotated DMPs were annotated to *PTGDS* and *PEX19* and showed elevated DNA methylation in plantation workers. Overall, 123 DMPs were identified in the blood samples, with the top two DMPs annotated to *WNT16* and *ENTPD8*, however, these only reached a suggestive significance level (*P* < 0.0001). The investigators also studied DMPs in PD cases in relation to levels of detectable organochlorines in the brain. They found 78 DMPs at the same suggestive significance levels. However, these findings were not sufficiently adjusted for the genome-wide multiple-testing burden.

Both epigenetic studies of pesticide exposure in PD followed up with pathway analyses. Paul et al. found that differential DNA methylation of the muscarinic acetylcholine receptor 1 and 3 pathways was enriched in response to pesticide exposure. This likely relates to the direct inhibition of acetylcholinesterase (AchE) activity by organophosphates^117^. Meanwhile, the top pathways enriched amongst DMPs in blood and brain for organochlorine (OGC) pesticide exposure were related to neurological disease, inflammatory response, and nervous system development^113^.

### Lifestyle factors

This final category encompasses any exposures falling broadly under the umbrella term ‘lifestyle factors’. Coffee and cigarette smoking have frequently been reported to have an inverse correlation with PD risk^53,118^. One epigenome-wide association study^119^ profiled DNA methylation across genes associated with coffee consumption and found one differentially methylated CpG site at Bonferroni-corrected significance level, annotated to *ALPPL2*, in blood samples profiled in the PEG1 and WHI control cohorts. Ten additional CpGs surpassed the *P* < 5.0 × 10^−6^ threshold: these were annotated to the genes *GPR132, BSCL2, GRK5, FSTL5, PTHLH, EIF1, KRT42P, MALRD1, PSMD8, TMEM130, FLJ22536, PODXL, CNTN4* and *ROBO3*. When adjusted for smoking, the association between coffee consumption and the 11 top-ranked CpGs remained. No significant associations were identified in saliva of PD or control participants (*n* = 127 cases, *n* = 215 controls).

Searles Nielsen et al.^120^ reported no association between the transposable DNA element LINE-1 methylation and PD in PBMCs. The authors did, however, report an association between ever smoking tobacco and reduced risk of PD. This association appeared to be stronger in men and women with the lowest LINE-1 methylation, but the interaction effect was not statistically significant (*P* = 0.06; *n* = 292 cases, *n* = 401 controls).

One EWAS^45^ investigated DNA methylation associated with PD progression. Of relevance to this review, the authors compared DNA methylation in whole blood of PD patients on dopamine replacement therapies to those not taking any PD medication (*n* = 27 cases, *n* = 162 controls). They reported differential methylation at 237 probes (*P* < 1.00 × 10^−7^) in PD participants taking medication, of which 23 CpGs were also associated with ageing. These changes were in the range of 0.63% reduction to 0.86% increase in DNA methylation per year. Genes annotated to these CpGs have functions in neural cell adhesion and synaptic transmission. PD participants not taking any medication showed 24 differentially methylated CpGs when compared to healthy controls. Notably, this group showed the largest effect sizes of any group, with differential methylation ranging from a 1.5% reduction to a 1.7% increase per year. These CpGs mapped to genes primarily involved in cytoskeletal function and immune response.

Dietary vitamin E has been suggested to be a protective factor in PD^121^. One candidate gene study^111^ looked at methylation across the *MAPT* gene, coding for microtubule-associated protein tau—a major susceptibility locus for idiopathic PD, in relation to PD and vitamin E exposure. Of interest, the authors noted taking levodopa was significantly inversely correlated with *MAPT* methylation in leucocytes of PD patients (*P* < 0.004; *n* = 1081 cases, *n* = 358 controls). The study also examined blood vitamin E levels and reported an association with *MAPT* methylation levels in control participants (*P* = 0.018), however, this association was not tested in PD samples.

Finally, a study by Oliveira and colleagues^112^ measured histone H4 acetylation in blood of PD patients. The study aimed to investigate whether a 4-week aquatic exercise program with or without supplementary grape juice influenced H4 acetylation in PD participants. The authors observed a global increase in H4 acetylation for participants (*n* = 19 cases, *n* = 10 exercise only, *n* = 9 exercise plus grape juice) in both exercise conditions (*P* = 0.031). No differences between the exercise alone and exercise plus grape juice groups were reported.

## Discussion

### Overall summary of findings

The notion of environmental risk factors implicated in PD is well-established and several studies have shown that epigenetic mechanisms can be altered in disease. However, epidemiological studies of environmental exposures in relation to epigenetic patterns in PD are only beginning to emerge. We identified studies investigating three main types of exposures: heavy metals, pesticides, or lifestyle factors and their associations with epigenetic variation in PD. The ten reviewed papers focused primarily on DNA methylation using surrogate tissues. Based on the included studies, there is suggestive evidence that pesticide exposure is associated with altered epigenetic marks, specifically DNA methylation, in PD cases. However, the two EWAS focussing on pesticides showed no overlap in differentially methylated CpG sites, possibly due to differences in the specific types of pesticides investigated: organophosphates^117^ versus organochlorines^113^. The other four EWAS all investigated different exposures and identified non-overlapping sets of DMPs, so there has been no attempt at replication yet. The sample sizes of EWAS ranged from small (*n* = 4) to moderate (*n* = 959) in size, and subsequently power to detect effects was variable. Half of the reviewed EWAS analysed the PEG cohort to find associations. Multiple testing correction was rarely performed to an adequate level, instead reporting “suggestive” significant results, which will require stringent independent replication. As it stands, the existing EWAS serve as a good starting point, but necessitate follow-up in-depth investigation adhering to more stringent methodological planning (see “Future directions”). Of the studies investigating global levels of epigenetic modifications, one measured global H4 acetylation and the other DNA methylation. Both studies were small, ranging from 10 to 97 cases. These studies did not sufficiently consider covariates, potentially increasing the risk of bias. Both candidate gene studies reviewed here came to conclusions about the effect of lifestyle factors on methylation of genes. However, neither study took into consideration cellular composition.

The quality of research on this topic so far is limited, owing to insufficient sample sizes, lack of independent replication of results and failure to investigate disease-relevant tissues and cell types. Therefore, no definitive conclusions could be drawn for any of the exposure categories due to a lack of overlap and replication of findings within the field. This does not, however, demonstrate the absence of an association, and the lack of consistency is likely to result in part from each study employing different approaches for assessing DNA methylation.

### Future directions

The major flaws and drawbacks identified in current literature on epigenetics involved in environmental exposures in PD were common across studies. Most of them relate to study design and statistical analyses. Due to overarching criticisms, we have specified some areas which future research should focus on. These are discussed below.

The specific approach taken to link epigenetic variation with environmental exposures can have an impact on the biological insights gained. Genome-wide approaches, covering the whole genome or at least an unbiased subset of regions spanning all genes and relevant regulatory elements, are the current gold standard for epidemiological studies. Conversely, a candidate gene approach narrows the field of discovery considerably, meaning potentially relevant genomic regions are ignored. We would argue that global studies of DNA methylation also have limited utility as epidemiological epigenetic studies as they lack specificity to implicate precise genomic mechanisms involved in the development of PD. For epigenome-wide association studies, the choice of methylation array is an important consideration. The majority of studies focused on DNA methylation using 450 K Bead-Chip Arrays. The more recent EPIC array nearly doubles the number of CpG sites covered to 850k, including more probes in non-coding regions and gene regulatory elements^122^. Both the 450 K and EPIC arrays rely on bisulfite treatment to convert unmethylated cytosine residues to uracil, allowing for the identification of methylated cytosine^123^. This method cannot differentiate between 5mC and 5hmC, however. Newer methods, such as oxidative bisulfite sequencing^124^ and nanopore sequencing^125^ are able to distinguish between the two marks. This is important, as 5mC and 5hmC can have different effects on gene regulation: 5mC at CpG islands is associated with reduced transcription, while 5hmC in intergenic regions is linked to increased gene expression^126^. It is also worth noting that DNA methylation is only one type of epigenetic modification, hence efforts should be made to investigate further epigenetic mechanisms, including histone modifications and non-coding RNA. Expanding research into less frequently profiled environmental exposures would be of interest to the field. For example, dairy consumption and plasma urate levels have been associated with PD risk, but no epigenetic studies have been conducted in humans yet^87,127^. Future work will also benefit from more comprehensively exploring the exposome^128^ and epigenetic signatures associated with it, providing insights into the cumulative effects of multiple exposures and their collective contribution to disease development and progression.

To produce accurate and reproducible findings, sample sizes need to be increased to improve power to detect effects. This is especially important in complex disease, where effect sizes may be small^129^. Therefore prior to beginning a study, power calculations should be carried out where possible to estimate required sample sizes. Literature and software to guide such power calculations have been reported previously for DNA methylation arrays and could, for example, be adapted from existing methods for RNA-seq in the case of histone modifications^130–132^ and non-coding RNAs^133–135^. Obtaining high-quality samples of well-characterized brain tissue can be exceedingly challenging, however. In this regard, leveraging existing samples from brain banking, clinical sources and multi-centre collaborations as well as future meta-analyses will be critical. Several cohorts, including those from PEG, were used across multiple studies. This could result in unwanted correlations and biases between studies. Therefore, establishing new cohorts is of interest to the field, to facilitate independent replication and increase sample sizes. When setting up these cohorts, extensive phenotypic data should be collected and included as covariates in analyses. Covariates that are known to affect epigenetic marks should be collected where possible, including age^136^, smoking status^137,138^, body mass index (BMI)^139,140^, alcohol use^141,142^, exposure to a range of environmental toxicants^143,144^, medications^145,146^, comorbid diseases^147,148^ and exercise regimen^149^. However, establishing a new cohort can take years before sufficient samples are collected, therefore incorporating existing brain bank data with already collected data from other fields, such as agricultural surveys, holds great potential in furthering our knowledge of how certain exposures can affect PD. An example of this would be to integrate data from projects such as US National Water-Quality Assessment^150^, which maps pesticide use by region and year. This has successfully been demonstrated in studies researching PD mortality^151^, but is yet to be linked to epigenetic profiles. Sensors to measure air quality are already in place worldwide, but novel methods to quantify the dose of specific air pollutants are still in early stages^152^. The use of such methods has large-scale, significant potential to uncover links between exposures and PD risk, and, when used in conjunction with detailed patient history in brain banks, can yield a better understanding of the role of epigenetic mechanisms in PD. There is also an important argument for streamlining future tissue donation for large and detailed population-based cohorts, such as UK Biobank^153,154^. This would enable researchers to link longitudinal clinical, phenotypic and survey data to post-mortem neuropathology and gene regulatory variation. At the minimum, information collected from donors of existing brain banks and clinical resources could be expanded to include survey data on PD relevant exposures and lifestyle factors. Epigenetic patterns identified in primary human post-mortem brain tissue could also be compared and integrated with data from in vivo and in vitro model systems, which allow for controlled administration of relevant exposures.

An important confounder specific to PD is the use of dopamine replacement medication. As reported previously by Henderson-Smith and colleagues^45^, anti-parkinsonian therapy has an effect on DNA methylation, which may place limitations on ideal study design. While adding further variables may reduce the power to detect associations, it is important to establish true, unbiased associations, particularly in complex disease where several small effects may contribute to the disease. Confounders may also act to add noise to true signal, therefore removing these can increase effect sizes. Variable selection methods can be used to determine which covariates should be included in analysis^155^.

To further increase the accuracy of studies, researchers should aim to sample tissue relevant to the disorder itself. In the case of PD, it is of great interest to measure epigenetic marks in disease-affected brain tissue, especially SNpc. While epigenetic profiles measured in peripheral tissues such as whole blood or saliva may hold utility for biomarker research, they are unlikely to yield causal biological insights into disease mechanisms. Evidence has indicated that epigenetic marks are not generally transferable from blood or saliva to disease-relevant cell types, necessitating brain-specific studies of PD^117,156,157^. This is a specific challenge to research in PD and other brain-related disorders, as generally these tissues can only be collected post-mortem. Recent evidence additionally suggests an important role for the enteric nervous system in PD^20,158^, alongside reports of α-synuclein in enteric ganglia cells^159,160^and skin^161^ samples. These tissues could be much more readily accessible via gut or skin biopsies and could prove very valuable in understanding environmental effects on gene regulation in disease-relevant cell types. There may also be utility in collecting more closely related surrogate tissue, which is likely to contain disease-relevant cells. This could include cerebrospinal fluid (CSF) samples, collected via lumbar puncture or from the brain, for example, collected during the treatment of hydrocephalus. Brain tissue resections offer a unique source of fresh brain tissue, although these are often limited to children and adolescents and therefore unlikely to overlap PD-affected populations. When working with complex tissue such as whole-brain or blood samples, it is essential to correct for cellular heterogeneity. The composition of these tissues is closely associated with epigenetic differences, given that epigenetic profiles are highly cell-type specific. Cell-type composition can differ between cases and controls, particularly in neurodegenerative diseases. If left unaccounted for, variation attributed to disease may in actuality be caused by differences in cellular composition^162^. Cell-type deconvolution approaches based on reference profiles can help account for heterogeneity and have been developed for bulk DNA methylation^163–165^, gene expression^166,167^, open chromatin^168^ and histone modification^169^ profiles. There are also laboratory techniques available to purify cells for cell-type specific epigenetic measures, such as fluorescence-activated cell sorting (FACS), which allows the sorting of bulk tissue into multiple cell types for omics interrogation^170^. Novel high-throughput single-cell approaches, including RNA^171^ and bisulphite sequencing^172^, as well as assay for transposase-accessible chromatin (ATAC)^173^ and Cleavage Under Targets and Tagmentation (CUT&Tag)^174^ allow transcriptomic and epigenomic profiling of low-input heterogeneous tissue at single-cell or -nucleus resolution.

Finally, to generate accurate and robustly significant results, proper implementation of statistical methods should be ensured. This includes basic issues, such as using appropriate methods to adjust for multiple testing. Some of the discussed studies discarded Bonferroni correction as overly stringent and agnostic to pairwise correlation of CpGs, opting instead for statistically unjustified lenient significance thresholds. Microarray-wide thresholds which take into account the correlation structure of CpG sites have been published and should be employed in these cases^175^. Implementation of these recommendations will lead to more robust and reliable study results and help unravel the intricate influence of individual environmental factors on epigenetic marks in Parkinson’s disease.

## Conclusions

By performing an extensive analysis of the published literature, we showed that promising results have been reported in in vitro models of PD, particularly following exposure to pesticides or metals in dopamine expressing cells. The existing literature linking exposures, epigenetic regulation and disease outcomes in humans is still very limited, but suggestive evidence of the effects of pesticide and metal exposure motivates further research. Importantly, we have identified a significant gap in the current knowledge regarding the effects of environmental exposures on epigenetic mechanisms in PD in humans. This provides an exciting opportunity as well as an urgent need for future research, linking environmental exposures and disease outcomes. We envisage that future studies with careful experimental design will elicit the role of epigenetic variation in response to environmental exposures in PD onset and progression. This would subsequently aid in determining functional mechanisms involved in exposure-disease associations and contribute to the development of targeted therapeutic approaches. Complementing this, non-functional disease-associated epigenetic variation from peripheral tissue will provide useful biomarkers for risk and diagnostic assessment.

### Reporting summary

Further information on research design is available in the Nature Research Reporting Summary linked to this article.

### Supplementary information

Supplementary S1

Supplementary Table 1

Supplementary Table 2

Reporting Summary
