# Supplementary material for: Linking environmental risk factors with epigenetic mechanisms in Parkinson’s disease
Source: NPJ Parkinsons Dis. 2023 Aug 25;9:123. doi: 10.1038/s41531-023-00568-z (PMC10457362; doi:10.1038/s41531-023-00568-z)
Supplement: Supplementary file 1 — Supplementary S1 [file 41531_2023_568_MOESM1_ESM.docx]

**Supplementary Data 1 -** List of search terms for different databases used for this review

**PubMed**

("Parkinson Disease"[mh] OR Parkinson*[tiab]) AND (Epigenomics[mh] OR epigenetics[tiab] OR "DNA methyl*" OR "CpG Islands"[mh] OR demethylat*[tiab] OR histone[tiab] OR "non-coding RNA") AND (toxicant[tiab] OR solvent[tiab] OR environment[tiab] OR cigarette*[tiab] OR smoking[tiab] OR coffee[tiab] OR caffeine[tiab] OR pesticide[tiab] OR metal[tiab] OR "air pollution" OR contaminant[tiab] OR exposure[tiab] OR ibuprofen[tiab])

**Embase/MEDLINE**

| 1. exp *Parkinson disease/ |
| --- |
| 2. Parkinson*.ti,ab,kw. |
| 3. exp *epigenetics/ |
| 4. epigenetics.ti,ab,kw. |
| 5. exp *DNA methylation/ or exp *DNA methyltransferase/ |
| 6. exp *CpG island/ |
| 7. demethylat*.ti,ab,kw. |
| 8. exp *histone/ or exp *histone modification/ |
| 9. "non-coding RNA".mp. or exp *untranslated RNA/ |
| 10. toxicant.ti,ab,kw. |
| 11. solvent.ti,ab,kw. |
| 12. exp *environment/ |
| 13. smoking.ti,ab,kw. |
| 14. coffee.ti,ab,kw. |
| 15. caffeine.ti,ab,kw. |
| 16. exp *pesticide/ |
| 17. metal.ti,ab,kw. |
| 18. exp *air pollution/ |
| 19. contaminant.ti,ab,kw. |
| 20. exposure.ti,ab,kw. |
| 21. ibuprofen.ti,ab,kw. |
| 22. 1 or 2 |
| 23. 3 or 4 or 5 or 6 or 7 or 8 or 9 |
| 24. 10 or 11 or 12 or 13 or 14 or 15 or 16 or 17 or 18 or 19 or 20 or 21 |
| 25. 22 and 23 and 24 |

**Web of Science**

(TS=(Parkinson*  AND (epigenomic*  OR epigenetic*  OR "DNA methyl*"  OR "CpG Islands"  OR demethylat*  OR histone  OR "non-coding RNA")  AND (toxicant  OR solvent  OR environment  OR cigarette*  OR smoking  OR coffee  OR caffeine  OR pesticide  OR metalOR "air pollution"  OR contaminant  OR exposure  OR ibuprofen))) *AND*LANGUAGE: (English)

**SCOPUS**

TITLE-ABS-KEY ( parkinson*  AND  ( epigenomic*  OR  epigenetic*  OR  "DNA methyl*"  OR  "CpG Islands"  OR  demethylat*  OR  histone  OR  "non-coding RNA" )  AND  ( toxicant  OR  solvent  OR  environment  OR  cigarette*  OR  smoking  OR  coffee  OR  caffeine  OR  pesticide  OR  metal  OR  "air pollution"  OR  contaminant  OR  exposure  OR  ibuprofen ) )
